# False Teeth Perforating a True Lumen: A Story of a Denture Adventure With Hypopharyngeal Perforation

**DOI:** 10.14309/crj.0000000000002018

**Published:** 2026-02-20

**Authors:** Malique Delbrune, Thomas Enke, Mohammad Bilal

**Affiliations:** 1 Department of Internal Medicine, University of Colorado Anschutz Medical Campus, Aurora, CO; 2 Division of Gastroenterology and Hepatology, University of Colorado Anschutz Medical Campus, Aurora, CO

## CASE REPORT

**Figure 1.**
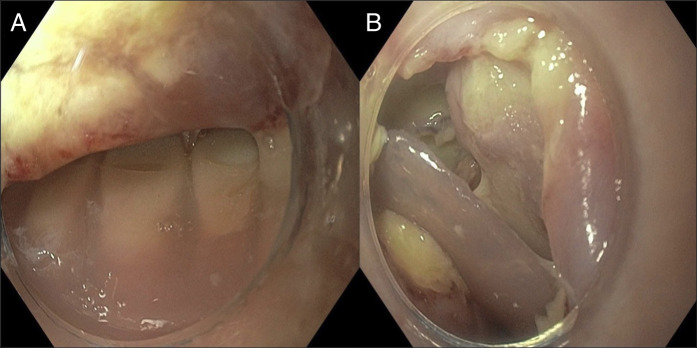
Partial denture embedded in the hypopharynx (A) and associated mucosal defect (B).

**Figure 2.**
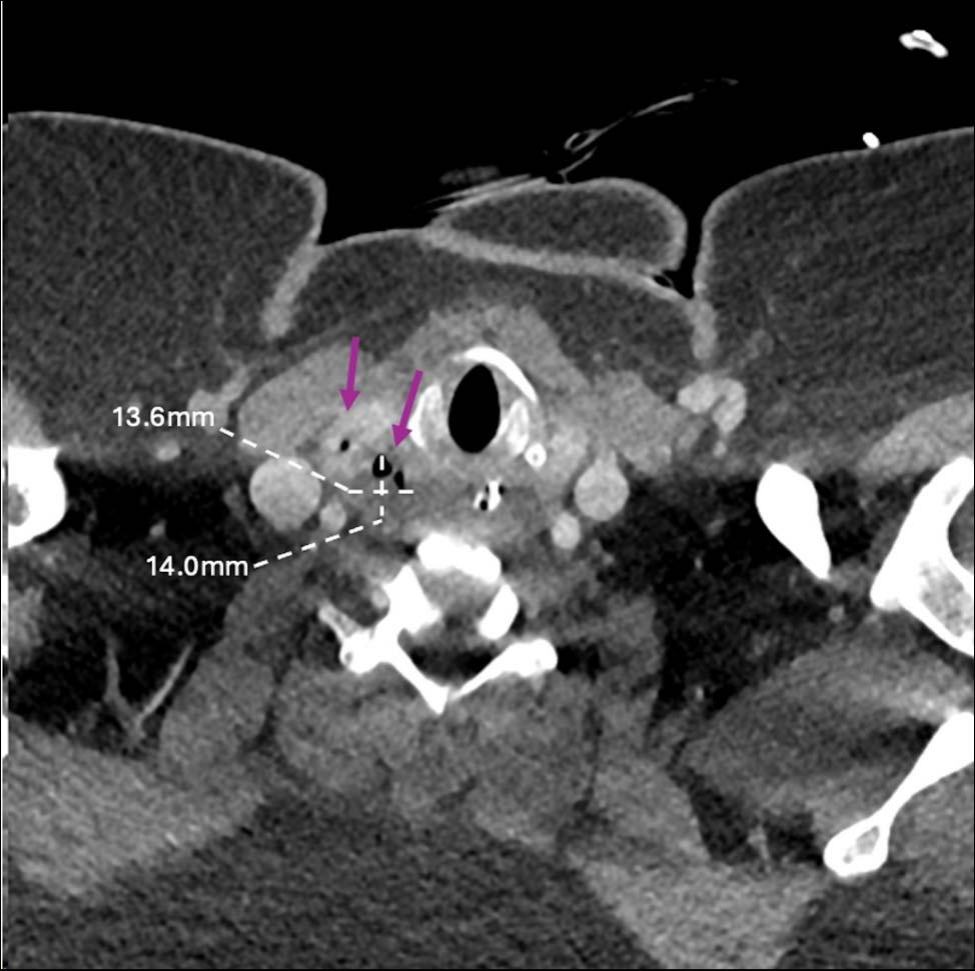
Computed tomography scan of neck with 1.36 cm × 1.4-cm sinus gas collection (purple arrows) between the right lateral margin of hypopharynx and superior thyroid.

**Figure 3.**
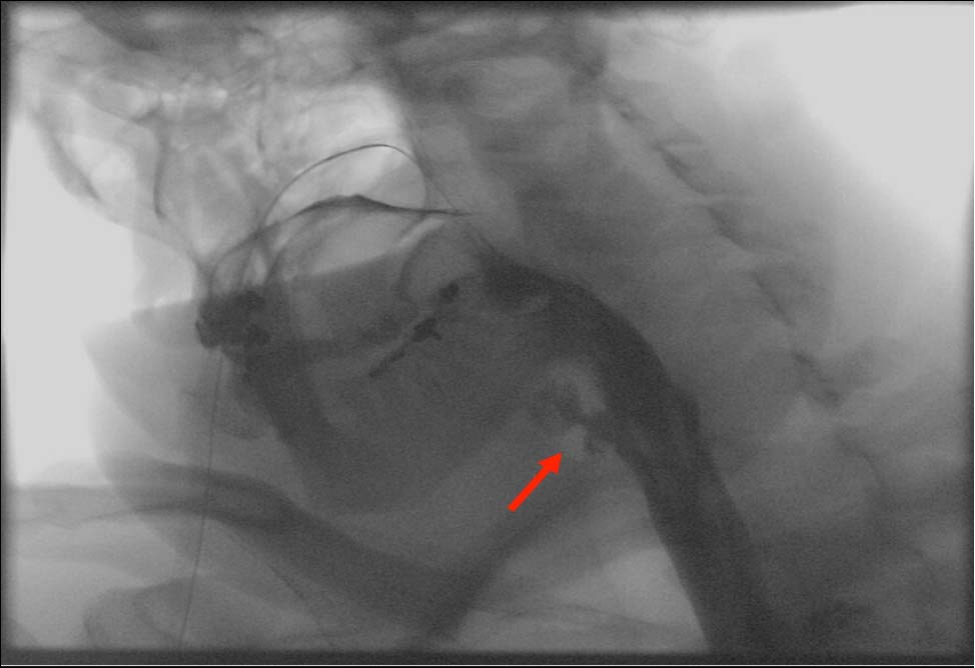
Esophagram demonstrating persistent leak (red arrow) from a hypopharyngeal perforation.

A 50-year-old woman presented with dysphagia, odynophagia, and dyspnea following accidental dentures ingestion. Computed tomography revealed upper esophageal and retropharyngeal soft tissue thickening. No radiopaque foreign body or perforation was visualized. Intravenous antibiotics were initiated. Esophagogastroduodenoscopy demonstrated an embedded denture with surrounding ulcerated mucosa in the hypopharynx (Figure 1). Removal was accomplished with foreign body hood and grasping forceps without visualization of full-thickness perforation. A nasogastric tube was placed endoscopically. She remained intubated and nil per os (NPO). Computed tomography neck on postoperative day (POD) 1 demonstrated gas extending from the hypopharynx into the thyroid with an associated 1.4-cm fluid collection (Figure 2). She was extubated POD 2. Esophagogastroduodenoscopy on POD 5 revealed ulcerated mucosa with granulation tissue. Esophagram on POD 6 revealed persistent leak from the hypopharynx (Figure 3). Conservative management, including antibiotics and tube feeds, was continued. Esophagram on POD 11 demonstrated no leak. Diet was advanced from clear liquids to general diet over 48 hours.

Foreign bodies resulting in perforation within the cervical esophagus and hypopharynx pose a unique challenge. Dentures are often radiolucent making identification difficult.^1^ Conservative management, with or without drainage, is frequently sufficient following prompt recognition in the absence of systemic toxicity or large defects.^2–4^

## DISCLOSURES

Author contributions: M. Delbrune: Primary manuscript writing; T. Enke: Revisions and endoscopic image acquisition; M. Bilal: Overseeing investigator who performed with endoscopy and ensured accuracy of technique for removal. M. Bilal is the article guarantor.

Financial disclosure: Dr Mohammad Bilal is a consultant for Boston Scientific, Steris Endoscopy, Aspero medical, and Cook endoscopy. There are no conflicts of interest to disclose in the creation or submission of this manuscript.

Previous presentation: Presented at American College of Gastroenterology Conference in Phoenix, AZ 2025.

Informed consent was obtained for this case report.
